# Metabolomics of Duodenal Juice for Biliary Tract Cancer Diagnosis

**DOI:** 10.3390/cancers15174370

**Published:** 2023-09-01

**Authors:** Kazuma Kishi, Masaki Kuwatani, Yuki Ohnishi, Yasuhiro Kumaki, Hiroyuki Kumeta, Hajime Hirata, Yunosuke Takishin, Ryutaro Furukawa, Kosuke Nagai, Hiroki Yonemura, Shunichiro Nozawa, Ryo Sugiura, Kazumichi Kawakubo, Tomoyasu Aizawa, Naoya Sakamoto

**Affiliations:** 1 Department of Gastroenterology and Hepatology, Hokkaido University Faculty of Medicine and Graduate School of Medicine, North 15, West 7, Sapporo 060-8648, Hokkaido, Japan; homakku@gmail.com (K.K.); h.hirapon@gmail.com (H.H.); ofmycloud@live.jp (Y.T.); rfurukawa404@gmail.com (R.F.); n.kosuke0620@gmail.com (K.N.); kyokui100055@gmail.com (H.Y.); shunichiro.nozawa@gmail.com (S.N.); ryou99sugi@yahoo.co.jp (R.S.); kkawakubo-gi@med.hokudai.ac.jp (K.K.); sakamoto@med.hokudai.ac.jp (N.S.); 2 Department of Advanced Transdisciplinary Science, Faculty of Advanced Life Science, Hokkaido University, Sapporo 060-0810, Hokkaido, Japan; yonishi@sci.hokudai.ac.jp (Y.O.); kumaki@sci.hokudai.ac.jp (Y.K.); kumetanmr@sci.hokudai.ac.jp (H.K.); aizawa@sci.hokudai.ac.jp (T.A.)

**Keywords:** metabolomics, duodenal juice, biliary disease, biliary tract cancer, cholangiocarcinoma

## Abstract

**Simple Summary:**

Biliary tract carcinoma (BTC) has an extremely poor prognosis, with a 5-year survival rate of less than 30%. However, the sensitivity and accuracy of current diagnostic tools, such as blood tumor marker and pathological diagnosis via endoscopic retrograde cholangiography (ERC), is low. Our study aimed to perform a metabolomic analysis of duodenal juice, which can be collected noninvasively, as a novel diagnostic method, and to investigate its added value as a biomarker for the diagnosis of BTC. We collected duodenal juice from 67 patients and performed metabolomic analysis prospectively. We found differences in the metabolome between malignant and benign biliary diseases. The metabolomic analysis of duodenal juice is feasible and available for differential diagnosis of biliary diseases’ malignancy and benignancy.

**Abstract:**

The poor prognosis of malignant biliary diseases is partially caused by their difficult early diagnosis. Therefore, many patients are only diagnosed at advanced stages. This study aimed to improve diagnosis by clarifying the differences in the duodenal juice metabolomes of benign and malignant biliary diseases. From October 2021 to January 2023, duodenal juice was obtained from 67 patients with suspected biliary diseases who required endoscopic ultrasonography and endoscopic retrograde cholangiography for diagnosis/treatment. The samples metabolomes were analyzed via nuclear magnet resonance spectroscopy using an 800-MHz spectrometer. Metabolomes of malignant and benign diseases were then compared, and multivariate analysis was performed to determine the relevant factors for malignancy/benignancy. For benignancy, no significant predictors were observed. For malignancy, acetone was a significant predictor, with higher concentrations in the malignant group than in the benign group. Regarding the receiver operating characteristic curve analysis for biliary tract carcinoma diagnosis, the predictive value of acetone in duodenal juice was comparable with serum CA19-9 levels (area under the curve: 0.7330 vs. 0.691, *p* = 0.697). In conclusion, duodenal juice metabolomics is a feasible method that is available for differential diagnosis in the biliary disease field.

## 1. Introduction

Biliary tract carcinoma (BTC) has an extremely poor prognosis, with 5-year survival rates of less than 30% [1]. As early diagnosis of BTC is difficult; many patients are only diagnosed at advanced stages and unresectable conditions [2]. Serum tumor markers such as CA19-9 are commonly used as biomarkers for biliary cancer diagnosis, with a reported sensitivity of 60% [3,4]. Furthermore, serum markers’ sensitivity can also be elevated in other cancers and benign diseases, such as breast cancer, lung cancer, or cholangitis. Although endoscopic retrograde cholangiography (ERC) allows pathological diagnosis of biliary disease via biopsy, its sensitivity and positive predictive value for BTC are 40–60% and 44–89%, respectively [5,6,7], which are insufficient for diagnosis. Despite advances in endoscopy, the percentage of adverse events associated with ERC, such as pancreatitis, remains at 10–12% [8,9]. Thus, safer and easier diagnostic methods are warranted.

Currently, research on multi-omics, such as via studies using saliva, blood, and tissues to analyze genomes, proteins, and metabolomes and to search for biomarkers and disease causes, is being conducted [10]; the metabolome is located downstream in the gene products closest to the human phenome, which could be influenced by changes in the metabolome [10]. In addition, metabolomics has the advantage of easily analyzing metabolomes, as the number of target substances is small compared to those of genomics and transcriptomics [11]. Previous studies on metabolomics performed with blood and body fluids, such as urine, bile, and pancreatic juice, indicated that metabolomes can be indicators of disease-specific profiles [12,13,14,15]. Regarding blood metabolomics, Lenka et al. [16] reported that pancreatic cancer patients had predominantly higher levels of metabolites such as 3-hydroxybutyrate and mannose. As for bile metabolomics, Urman et al. [17] reported that valine and formate concentrations were significantly elevated in BTC patients, and acetate and phosphocholine in pancreatic cancer patients. Regarding metabolomics of pancreatic juice, Cortese et al. [18] reported a predominant increase in lactate in pancreatic cancer patients. There are few published reports of metabolomics on cancers in the pancreaticobiliary region, especially on biliary tract cancers. Furthermore, collecting bile and pancreatic juice by endoscopic retrograde cholangiopancreatography has a risk of adverse events such as pancreatitis and cholangitis [19]. On the other hand, duodenal juice includes bile and pancreatic juice and can be easily collected during esophagogastroduodenoscopy or endoscopic ultrasonography (EUS). Thus, our study focused on metabolomics of duodenal juice.

In duodenal juice analysis, Kisiel et al. [20] reported that methylated DNA is a promising early diagnostic marker of pancreatic cancer, while Matsunaga et al. [21] measured the levels of the protein marker S100P on duodenal juice and found that they could discriminate between pancreatic cancer patients and benign controls with 85% sensitivity and 77% specificity. However, duodenal juice analysis application has yet to reach daily clinical practice and is currently limited to experimental use.

There are two major methods for metabolome analysis: mass spectrometry (MS) and nuclear magnetic resonance (NMR). MS includes capillary electrophoresis–MS, gas chromatography–MS, and liquid chromatography–MS. MS has high sensitivity; however, it is difficult to identify a wide range of metabolomes via a single method, and it is complicated by several preprocessing steps, in addition to poor reproducibility. NMR, although less sensitive, can analyze a wide metabolome range by itself, and is considered more useful for identifying diagnostic markers due to its simplicity in preprocessing and high reproducibility [22].

Therefore, this study aimed to clarify the metabolome differences between biliary benign and malignant lesions via NMR metabolomics of duodenal juice as an effective and minimally invasive alternative diagnostic method for biliary diseases.

## 2. Materials and Methods

### 2.1. Patients

From October 2021 to January 2023, duodenal juice was prospectively collected from the participants registered at Hokkaido University Hospital. The inclusion criteria were as follows: (1) patients with suspected biliary lesions via serum biochemistry or imaging via ultrasonography/computed tomography/magnetic resonance who were scheduled to undergo esophagogastroduodenoscopy, EUS, ERC, or endoscopic ultrasonography-guided fine needle aspiration (EUS-FNA); and (2) those who provided written consent to participate in this study. Exclusion criteria were as follows: (1) patients who were unable to undergo oral or nasal endoscopic insertion; (2) patients who were deemed inappropriate as research subjects by the physician. All patients provided written informed consent for participation in this study, the analyses of their samples, and the use of their clinical data. Final diagnoses were based on the pathological assessment of forceps biopsy, brush abrasion, and surgical specimens by expert pathologists, and clinical courses. Malignancies were defined as follows: (1) pathological evidence using surgical specimens or cytology via bile/brush scrubbing; and (2) clinical and radiographic progression within ≥ 6 months such as enlargement of the mass, deterioration of biliary tract stricture, detection of metastatic disease, and elevation of serum tumor markers.

### 2.2. Sample Collection

When performing EUS, ERC, or EUS-FNA, the scope (ERC: TJF 260V; Olympus Medical Systems, Tokyo, Japan; EUS and EUS-FNA: UCT-260; Olympus Medical Systems, Tokyo, Japan) was inserted to the descending leg of the duodenum, and a catheter (PR130Q: Olympus) with a side hole was lightly pressed against the mucosa to collect duodenal juice accumulated around the Vater’s papilla. In order to exclude the effect of contamination of food or drugs as much as possible, we instructed patients to stop eating and taking medications before the examination, and, furthermore, we aspirated the gastric reservoir before collecting the duodenal fluid. After specimens were collected, they were preserved in blood collection tubes with aprotinin (LSI Medience corporation, Tokyo, Japan) to inhibit the activity of pancreatic enzymes and immediately stored at −30 °C until measurement. In ^1^H-NMR, trimethylsilyl propanoic acid (TSP) was used as the standard for chemical shifts. First, the sample was dissolved in water at 4 °C and centrifuged at 4 °C, 15,000 rpm for 15 min. Then, 700 μL of the supernatant was collected and carefully transferred to a 2 mL tube (Eppendorf manufacturing corporate, Enfield, CT, USA), and centrifugation was performed again at 4 °C, 15,000 rpm for 15 min. Then, 540 μL of the supernatant was carefully collected and carefully transferred to another tube, to which 60 μL of buffer (500 mM NaP: pH 7.4, 5 mM TSP, 100% D2O, 0.04% NaN3, 10 mM formate) was added. The tube was then centrifuged at 4 °C, 15,000 rpm for 15 min, and 550 μL of the supernatant was placed in a 5 mm NMR tube for measurement.

### 2.3. NMR Measurements and Analysis of Metabolomics Data

All spectra were acquired using an NMR spectrometer (Bruker Biospin Ascend 800 MHz) and the acquisition was set at 298 K. A one-dimensional NOESY pulse sequence was conducted, and the obtained spectrum was phase-matched using TopSpin 3.5 (Bruker Biospin, Rheinstetten, Germany). The TSP control area was fixed in the standard concentration using Chenomx NMR suite 9.0 (Spectral database; Edmonton, AB, Canada); then, metabolomes were identified and quantified. SIMCA-P version 17 (Umetrics, Umea, Sweden) was used for data analysis. Orthogonal projections to latent structure-discriminant analysis (OPLS-DA) were performed to create a discriminant model that maximally resolved the benign and malignant groups of this study. After that, the value of variable importance in the project (VIP) was measured and the contributing factors were explored. VIP refers to the contribution to the creation of the discriminant model of each variable, and metabolites with a VIP > 1 were considered as contributing metabolites; furthermore, the larger the VIP, the higher the contribution. For analyses for metabolite concentrations, the values were measured in a blinded manner regarding clinical information.

### 2.4. Statistical Analysis

All statistical analyses are performed with SIMCA-P 17 (Umetrics, Umea, Sweden) and free software EZR version 2.8-0 (accessed on 18 February 2023 at https://www.jichi.ac.jp/saitama-sct/SaitamaHP.files/statmedEN.html) [23]. Fisher’s exact test was performed to analyze categorical data. A *p*-value < 0.05 was considered statistically significant. The Mann–Whitney U test was used to compare the differences between the two groups. The receiver operating characteristic (ROC) analysis was based on logistic regression.

## 3. Results

### 3.1. Patient Characteristics

Sixty-seven eligible patients were enrolled in this study. The male to female ratio (M:F) was 34:3. In the malignant group, the median age was 73 years (35–96), and M:F was 21:12; in the benign group, median age was 64.5 years (27–86), and M:F was 13:21. The malignant group included 26 cholangiocarcinoma, 5 gallbladder cancer, and 2 ampullary carcinoma cases. Stage classifications were as follows: stage I, 3; II, 9; III, 12; IV, 9, according to the 8th edition of the UICC clinical staging. The benign group included 16 bile duct stone/gallbladder stones cases, 7 gallbladder polyp cases, 3 cases of adenomyomatosis of the gallbladder, 1 IgG4-related sclerosing cholangitis case, 1 chronic cholecystitis case, and 6 cases without imaging abnormalities. CA19-9 levels ranged from 2.1 to 167,959.3 U/mL (median, 101.3 U/mL) in the malignant group and 2.1 to 759.8 U/mL (median, 17.5 U/mL) in the benign group (*p*-value < 0.05) (Table 1). There were 18 cases with jaundice (malignant group 17; benign group 1), 5 cases with a biliary stent (5; 0), 10 cases with ursodeoxycholic acid treatment (1; 9), and only 1 case with HB virus infection (benign group).

### 3.2. NMR Spectra

**Figure 1:**
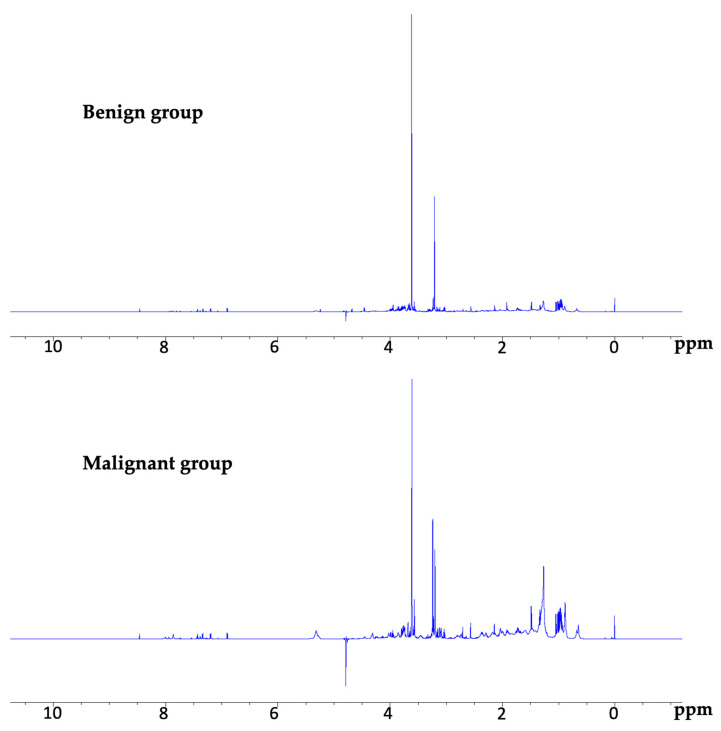
Examples of NMR spectra in the benign and malignant groups. The general spectral features were similar in both groups. NMR, nuclear magnetic resonance.

We obtained good NMR spectra for all duodenal juice samples from both groups. The general spectral features were similar, with large peaks in the aliphatic region (2.5–0.5 ppm) corresponding to the bile acids, cholesterol, fatty acids, and other lipid components (Figure 1).

### 3.3. Detection of Metabolites in the Malignant and Benign Groups

**Figure 2:**
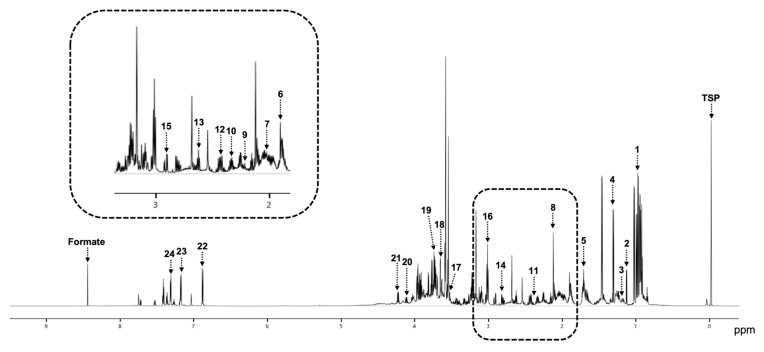
An example of resonances assignment in NMR spectra. 1. Isoleucine; 2. Propylene glycol; 3. 3-Hydroxybutyrate; 4. Lactate; 5. Leucine; 6. Acetate; 7. Valine; 8. Alanine; 9. Acetone; 10. Glutamate; 11. Pyruvate; 12. Glutamine; 13. Methionine; 14. Aspartate; 15. Asparagine; 16. Lysine; 17. Glycerol; 18. Ethanol; 19. Arginine; 20. Proline; 21. Threonine; 22. Tyrosine; 23. Tryptophan; 24. Phenylalanine. TSP, trimethylsilyl propanoic acid.

The metabolites were identified based on the combination of spikes (Figure 2). On average, 23 metabolites were detected from the NMR spectra of duodenal juice samples, and we were able to identify essential amino acids (e.g., valine, leucine, isoleucine, phenylalanine, threonine), nonessential amino acids (e.g., alanine, tyrosine, asparagine, glutamate), and ketone bodies (acetone and 3-hydroxybutyrate). The metabolites identified and their concentrations are listed from highest to lowest concentration in Table 2, with leucine having the highest concentration (1.33 mM) and acetone having the lowest concentration (0.03 mM). There was no difference in the type of metabolites (essential amino acids, nonessential amino acids, and ketone bodies) identified in the malignant and benign groups.

### 3.4. Statistical Analysis Using OPLS-DA

**Figure 3:**
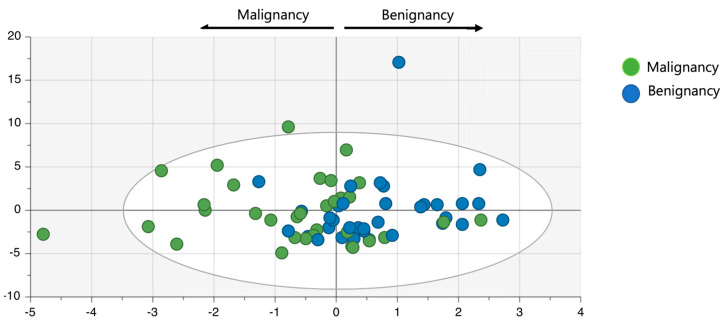
Statistical analysis with OPLS-DA. The data were given benign and malignant information and were statistically found to fall into two categories. OPLS-DA, orthogonal projections to latent structure-discriminant analysis.

**Figure 4:**
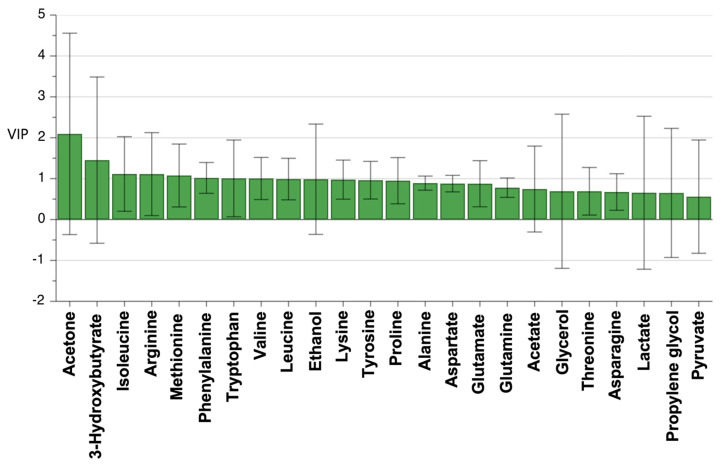
VIP values of metabolites. VIP values larger than 1.0 indicate important variables, which were acetone, 3-hydroxybutyrate, isoleucine, arginine, methionine, phenylalanine, tryptophan, and valine. Each bar with lines represents the mean with a 95% confidence interval. VIP, variable importance in the project.

**Figure 5:**
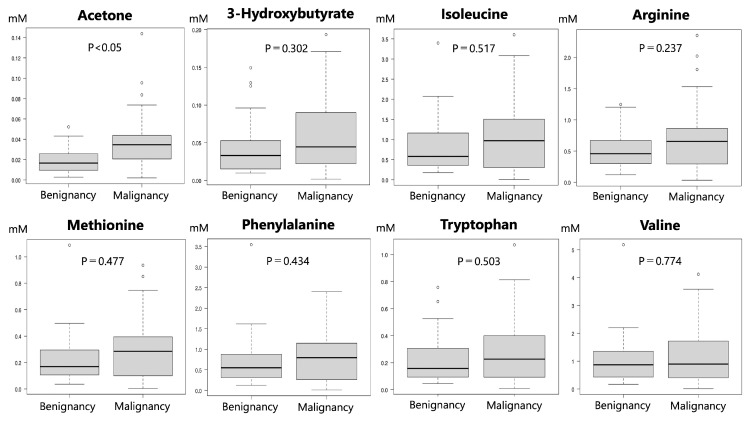
Concentrations of metabolites contributing to differentiation between the malignant and benign groups in OPLS-DA. The results showed that only acetone had a significantly higher concentration in the malignant group.

The OPLS-DA with information on the pathological or clinical diagnosis of a benign or malignant disease indicated that the data could fall into two categories according to the metabolites’ contributing factors (Figure 3). Furthermore, the VIP values of the metabolites identified showed that acetone, 3-hydroxybutyrate, isoleucine, arginine, methionine, phenylalanine, tryptophan, and valine contributed to discriminating malignancy from benignancy (Figure 4). When the respective concentrations of those eight metabolites in the benign and malignant groups were compared, acetone alone was significantly more abundant in the malignant group; meanwhile, no significant differences were observed for the other seven metabolites (Figure 5).

### 3.5. ROC Curves of CA19-9 and Acetone for a Diagnosis of Biliary Tract Cancer

**Figure 6:**
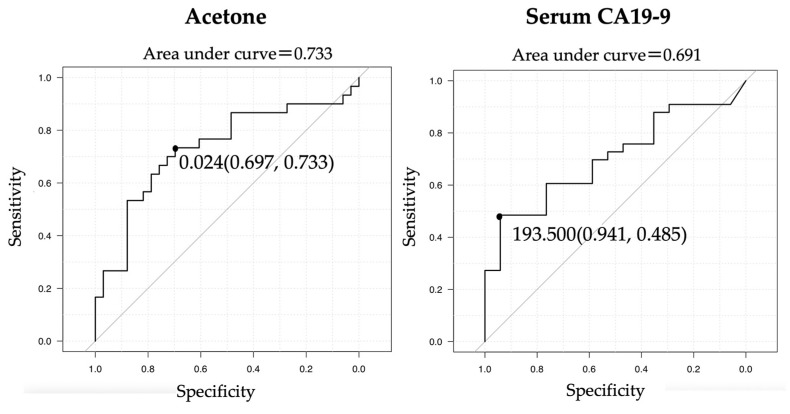
ROC curves of acetone and CA19-9. Acetone was comparable with serum CA19-9 for diagnosing BTC (AUC: 0.733 vs. 0.691, *p* = 0.697) if the cutoff value of acetone was set at 0.024 and that of CA19-9 was set at 193.5. ROC, receiver operating characteristic; BTC, biliary tract carcinoma; AUC, area under the ROC curve.

Based on the VIP scores and the metabolites concentration, we compared the diagnostic ability of acetone with that of serum CA19-9, which is the most common serum tumor maker in BTC, using a ROC curve analysis. In the ROC analysis, acetone was comparable with serum CA19-9 for diagnosing BTC (area under the ROC curve (AUC): 0.733 vs. 0.691, *p* = 0.697) if the cutoff values for acetone and CA19-9 were set at 0.024 mM and 193.5 U/mL, respectively. The sensitivity and specificity of acetone at its most favorable point were 69.7% and 73.3%, respectively, while those of CA19-9 were 94.1% and 48.5%, respectively. (Figure 6).

## 4. Discussion

This is the first report on the feasibility and availability of metabolomics of duodenal juice for differentiating malignancy from benignancy in the biliary tract field. 

There have been various reports regarding metabolomics for diagnosing biliary tract cancer, many of which used blood or bile as samples. Analysis of serum amino acid levels in patients with intrahepatic cholangiocarcinoma and healthy controls showed differences in some serum amino acid profiles, such as aspartic acid [24]. For bile samples, NMR-based metabolomics were reported as having a good performance in discriminating biliary tract cancer from benign biliary diseases [25]. In blood metabolomics, it is frequently difficult to determine where cancer is present, while direct bile collection via ERC has higher risks of adverse events such as pancreatitis and cholangitis. Therefore, metabolomics of duodenal juice presents significant advantages in terms of sampling easiness and safety. 

In addition, this study showed that the concentration of acetone was significantly higher in the malignant group than in the benign group. Ketone bodies (e.g., acetone and 3-hydroxybutyrate) have been reported to suppress tumor growth and improve cancer cachexia, probably as a biological defense response [26], although their role is not clear and is still being debated [27]. Other reports on serum metabolomics indicated that patients with pancreatic and prostate cancer had more acetone in their sera than healthy individuals [28,29,30]. In prostate cancer, ketone bodies, including acetone, were reported to play an important role in tumor growth and, although the detailed mechanism is unknown, a potential metabolic alteration may be involved in prostate cancer progression and metastasis [30]. It has also been reported that acetone gas release from the skin is increased in patients with pancreatic cancer compared to that in healthy controls [31]. In general, normal cells utilize ketone bodies as metabolic fuel during glucose starvation, but many tumor cells have been shown to utilize ketone bodies inefficiently due to mitochondrial dysfunction and ketone degrading enzymes deficiency [32,33]. Therefore, for biological defense, ketones are thought to be more abundant in malignant patients. Among ketone bodies, 3-hydroxybutyrate showed no significant difference between the groups but tended to be higher in the malignant group, which is also consistent with the previous findings described above [26]. The differences between acetone and 3-hydroxybutyrate may be explained by the fact that acetone, unlike 3-hydroxybutyrate, is not used for energy metabolism and is released directly into the blood [34]. Furthermore, the ROC analysis in the current study revealed that acetone was comparable with serum CA19-9 for BTC diagnosis. CA19-9 is a carbohydrate antigen consisting of sialic acid added to Lewis A, one of the blood group antigens; therefore, serum CA19-9 can be a false negative marker in the case of sialyl-Lewis-antigen-negative individuals, which account for 10% of the Japanese population [35]. Thus, acetone is an available and superior marker compared to serum CA19-9 in such cases. In blood metabolomics, it is reported that the sensitivity ranged between 55 and 95% and specificity between 58 and 100%, while bile metabolomics present a sensitivity of 88% and a specificity of 81% using an NMR-based metabolomics approach [24,25]. In the present study, when the cutoff value of acetone was 0.024 mM, the sensitivity and specificity were 69.7% and 73.3%, respectively, showing comparable diagnostic performance with those of blood and bile. In addition, it has been reported that simply measuring bile could result in a poor spectrum, and that the addition of an organic solvent during preparation is required [36]; while, in the analysis of duodenal fluid, a good spectrum was obtained without any special pretreatment. The obtention of a spectrum with less aggregation in duodenal fluid might result from an influx of bile and pancreatic juice into the duodenum, which dilutes the bile component and makes it easier to obtain an NMR spectrum. Furthermore, acetone, which was detectable in the current study, is volatilized in preparation by adding organic solvent; thus, it cannot be detected by other methods such as MS.

Although not significant, a higher propensity for malignancy was observed in the remaining six contributing metabolites: valine, isoleucine, arginine, methionine, phenylalanine, and tryptophan. Valine and isoleucine are classified as branched-chain amino acids, whose uptake by cancer cells tends to be increased due to the higher expression of aminotransferases in such cells [37]. Arginine is a semi-essential amino acid, but it becomes a fully essential amino acid in many cancers as it is a precursor for polyamines and nucleic acids and essential for protein synthesis. Furthermore, arginine cannot be metabolized in cancer cells due to loss of function of argininosuccinate synthase 1; therefore, arginine may be accumulated in cancer cells without being used [38]. Methionine and phenylalanine are essential amino acids that play an important role in the growth and metabolism of cancer cells [39,40]. In actively proliferating cancer cells, tryptophan metabolism is enhanced [41]. For cancer cells to survive, proliferate, and metastasize, for example, they must secrete growth factors that will act in an autocrine or paracrine manner or communicate with other cells. Recently, it has been reported that cancer cells secrete kynurenine, a tryptophan metabolite, which is a strategy that enables survival and proliferation, and which may be subsequently reflected in changes in tryptophan levels in duodenal juice.

This study has several limitations. First, the benign controls contained few healthy subjects. Therefore, the difference between malignancy and the healthy condition is unclear. Thus, the comparison between BTC and healthy controls should be assessed in a large-scale study that aims to apply this method to medical checkups. Second, we did not directly compare the diagnostic ability of duodenal juice with those of bile, blood, and urine in our cohort. For accurate assessment of their difference, direct comparison in the same cohort is necessary. Third, we were not able to examine whether there were differences in metabolites in different cancer stages, different cancer sites, or whether they are useful for detecting early-stage cancer because the sample size was small, and early-stage cancers were not included in this study. Fourth, we could not examine the change of metabolites according to patient characteristics; for example, with/without biliary stent, ursodeoxycholic acid treatment, and hepatitis virus (e.g., HBV or HCV). Biliary stents can increase the influx of bile into the duodenum, which might result in changes in metabolism in the duodenal juice. Previous reports indicate that amino acid metabolism could be changed with the use of ursodeoxycholic acid and the presence of hepatitis virus [42,43]. Although the sample size of our cohort was small, since there has been no report regarding metabolomics of duodenal juice, we could not set an appropriate sample size based on a proper assumption or pivotal evaluation of the accuracy of a biomarker [44]. Thus, we enrolled patients with a suspicious biliary disease from October 2021 to January 2023 whenever possible. Simultaneously, we also enrolled patients with a suspicious pancreatic disease and obtained similar results, which suggested acetone as a significant metabolome for pancreatic cancer (supplement Table S1 and Figures S1 and S2). Furthermore, based on the data on the total cohort with pancreaticobiliary diseases, we also obtained results which suggested acetone as a significant metabolome for malignancy (supplement Figures S3 and S4). When the cohort was divided into the two sub-cohorts, namely, the biliary and pancreatic disease groups, each cohort similarly showed that acetone was a significant metabolome for malignancy; that is, the metabolomics of duodenal juice derived from patients with pancreaticobiliary malignancy showed a similar result regardless of the lesion site. Fifth, we could neither analyze the metabolic pathways nor perform multi-omics in this study. To investigate and discuss the metabolome in malignant diseases, a one-time snapshot metabolomics analysis of only 20 metabolites might be insufficient. Meanwhile, in this study, we aimed to investigate the feasibility and availability of duodenal juice for metabolomics using NMR, and we verified both for the first time. 

In clinical practice, metabolomics of duodenal juice would provide a complementary diagnosis method in cases where cytology of bile and biopsy of the bile ducts provide indeterminate results.

## 5. Conclusions

Metabolomics of duodenal juice is a feasible and available method for the differential diagnosis of biliary malignancy and benignancy. Acetone in duodenal juice could be a useful indicator of a malignant biliary disease.

## Figures and Tables

**Table 1:** Patient characteristics.

	Malignant Group (N = 33)	Benign Group (N = 34)
Age, median (range), y	73 (35–96)	64.5 (27–86)
Sex, n (%)		
Male	21 (63.6)	13 (38.2)
Female	12 (36.4)	21 (61.8)
Details of disease, n (%)		
Cholangiocarcinoma	26 (78.7)	
Gallbladder cancer	5 (15.2)	—
Ampullary carcinoma	2 (6.1)	
Bile duct stone/gallbladder stone		16 (47.2)
Gallbladder polyp		7 (20.6)
No abnormality in workup imaging	—	6 (17.6)
Adenomyomatosis of gallbladder		3 (8.8)
IgG4-related disease		1 (2.9)
Chronic cholecystitis		1 (2.9)
UICC stage (*n* = 33), n (%)		
I	3 (9.2)	
II	9 (27.2)	—
Ⅲ	12 (36.4)	—
Ⅳ	9 (27.2)	
CA19-9, median (range), U/mL	101.3 (2.1–167,959.3)	17.5 (2.1–759.8)

**Table 2:** Metabolites identified.

No	Metabolite	Average Concentration (mM)
1	Leucine	1.33
2	Alanine	1.11
3	Valine	1.09
4	Isoleucine	0.92
5	Glycerol	0.87
6	Lysine	0.85
7	Phenylalanine	0.73
8	Ethanol	0.68
9	Tyrosine	0.66
10	Arginine	0.63
11	Proline	0.57
12	Threonine	0.53
13	Glutamine	0.52
14	Glutamate	0.42
15	Asparagine	0.33
16	Methionine	0.26
17	Tryptophan	0.25
18	Propylene glycol	0.20
19	Aspartate	0.20
20	Lactate	0.19
21	Acetate	0.19
22	3-Hydroxubutyrate	0.05
23	Pyruvate	0.03
24	Acetone	0.03

## Data Availability

The data presented in this study are available on request from the corresponding author. The data are not publicly available due to privacy laws.

## Supplementary Materials

The following supporting information can be downloaded at https://www.mdpi.com/article/10.3390/cancers15174370/s1; Table S1: ^1^H-NMR components assignment based on Chenomx Profiler at 800 MHz. Figure S1: To analyze the NMR data and to establish the prediction model for pancreatic malignant disease, we applied OPLS-DA multivariate analysis to the NMR data; Figure S2: VIP-values larger than 1.0 indicate important, which were acetone, 3-hydroxybutyrate, phenylalanine, tryptophan, proline, and propylene glycol; Figure S3: To analyze the NMR data and to establish the prediction model for pancreaticobiliary malignant disease, we applied OPLS-DA multivariate analysis to the NMR data; Figure S4: VIP-values larger than 1.0 indicate important, which were acetone and 3-hydroxybutyrate. Each bar with lines represents the mean with a 95% confidence interval.
