# Supplementary material for: Metabolomics of Duodenal Juice for Biliary Tract Cancer Diagnosis
Source: Cancers (Basel). 2023 Sep 1;15(17):4370. doi: 10.3390/cancers15174370 (PMC10486759; doi:10.3390/cancers15174370)
Supplement: Supplementary file 1 [file cancers-15-04370-s001.zip › cancers-2498654-supplementary.pdf]

Supplement Table S1

Patient characteristics	
Age, median (range), y	70 (27-86)
Sex, n (%)	
Male	52 (51)
Female	50 (49)
Details of disease, n (%)	
<u>Malignant disease</u>	
Pancreatic ductal adenocarcinoma	46 (45.1)
Intraductal papillary mucinous carcinoma	2 (2)
Metastatic pancreatic cancer	1 (1)
<u>Benign disease</u>	
Intraductal papillary mucinous neoplasm	19 (18.7)
Pancreatic cyst	10 (9.8)
Acute/Chronic pancreatitis	7 (6.8)
Neuroendocrine tumor	7 (6.8)
No abnormality in workup imaging	7 (6.8)
IgG4-related disease	3 (3)
CA19-9, median (range) U/ml	33.5 (2.1-32882161)

Supplement Figure S1

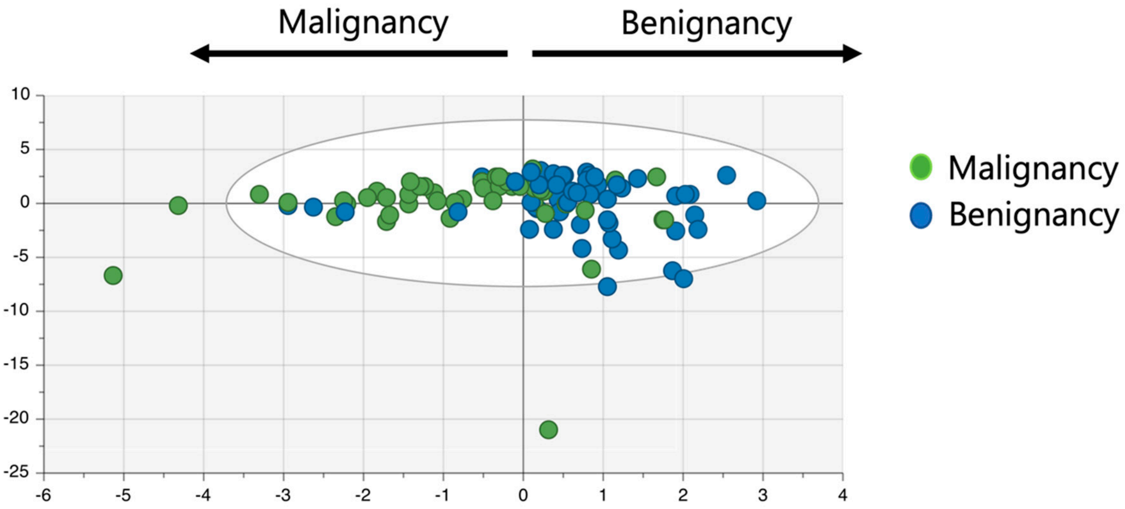

To analyze the NMR data and to establish the prediction model for pancreatic malignant disease, we applied OPLS-DA multivariate analysis to the NMR data. The majority of the benign and malignant samples appear clustered in their respective regions with only a few overlaps between them.

Supplement Figure S2

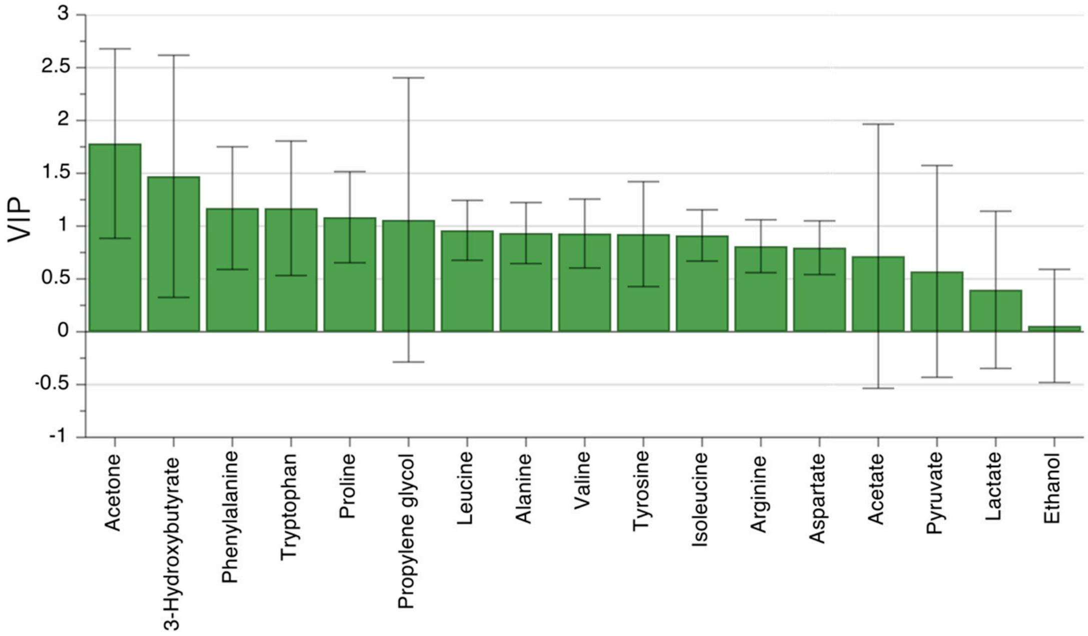

VIP-values larger than 1.0 indicate important, which were acetone, 3-hydroxybutyrate, phenylalanine, tryptophan, proline, and propylene glycol. Each bar with lines represents the mean with a 95% confidence interval. VIP, variable importance in the project.

Supplement Figure S3

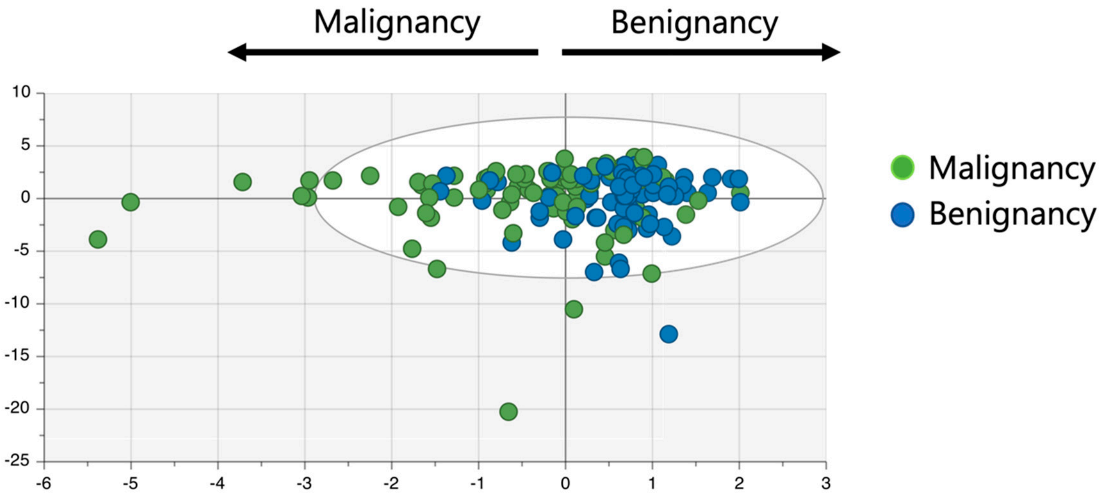

To analyze the NMR data and to establish the prediction model for pancreaticobiliary malignant disease, we applied OPLS-DA multivariate analysis to the NMR data. The majority of the benign and malignant samples appear clustered in their respective regions with only a few overlaps between them.

Supplement Figure S4

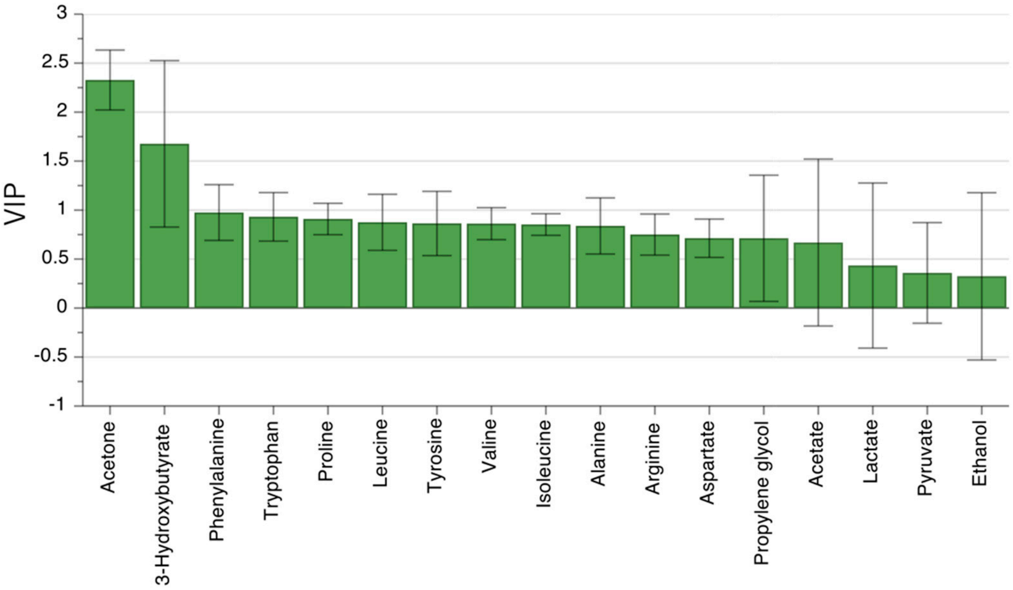

VIP-values larger than 1.0 indicate important, which were acetone and 3-hydroxybutyrate. Each bar with lines represents the mean with a 95% confidence interval. VIP, variable importance in the project.
